# Decoupling sporulation from insecticidal activity in *Bacillus thuringiensis* BLB1 strain through Iterative UV mutagenesis

**DOI:** 10.3389/fmicb.2026.1824913

**Published:** 2026-05-26

**Authors:** Fatma El Abed, Sameh Sellami, Baltasar Escriche, Souad Rouis

**Affiliations:** 1 Laboratory of Biotechnological Pest Control (CBP), Institute of Biotechnology and Biomedicine, University of Valencia, Valencia, Spain; 2 Laboratory of Biopesticides, Centre of Biotechnology of Sfax, University of Sfax, Sfax, Tunisia

**Keywords:** *Bacillus thuringiensis*, biological control, Iterative UV-mutagenesis, oligosporogenic mutants, whole genome sequencing

## Abstract

**Introduction:**

The persistence of *Bacillus thuringiensis* (*B. thuringiensis*) spores in the environment can lead to ecological and safety concerns, despite the widespread use of this microbial biopesticide. Developing oligosporogenic strains that retain high insecticidal activity represents a promising key strategy for sustainable pest management.

**Objectives:**

The aim of this study was to generate and characterize novel oligosporogenic mutants of the *B. thuringiensis* BLB1 strain through an iterative UV-mutagenesis approach, combined with Whole Genome Sequencing (WGS) to resolve the genetic basis of their phenotype.

**Methods:**

Successive sequential rounds of UV irradiation (254 nm) were applied, and mutants were screened for reduced sporulation while maintaining crystal production. Two promising candidates, T3 and T8, were selected for kinetic studies, protein quantification, and bioassays against five major lepidopteran pests. Their genomes were sequenced using Illumina technology and subjected to comparative genomic analysis.

**Results:**

Both mutants exhibited a significant delay in sporulation and altered glucose consumption patterns compared to the wild-type BLB1 strain. Despite the reduction in spore counts (5 × 10^7^ and 3 × 10^7^ spores/ml, respectively), T3 and T8 retained stable Cry protein profiles (Cry1 and Cry2). Bioassays revealed that both mutants maintained high toxicity, reflected by low LC50, particularly against *Grapholita molesta* (17 ng/cm^2^ for both mutants) and *Ostrinia nubilalis* (32 and 26 ng/cm^2^, respectively), compared to the wild-type strain BLB1 (25 and 18 ng/cm^2^, respectively). WGS confirmed that none of the detected *cry* genes were affected by mutagenesis, explaining the preserved insecticidal activity. These findings demonstrate that iterative UV mutagenesis effectively decoupled sporulation from insecticidal activity.

**Conclusion:**

These findings demonstrate that iterative UV mutagenesis effectively decoupled sporulation from insecticidal activity. The resulting mutants, T3 and T8, represent promising candidates for safer and more effective commercial biopesticide applications, combining reduced environmental persistence with strong biocontrol efficacy.

## 1 Introduction

Insect pests and plant pathogens represent a major threat to sustainable agriculture, due to significant crop damage and the reduced quality worldwide (Oerke, 2006). The excessive use of chemical pesticides has raised serious concerns regarding human health as well as environmental integrity. Indeed, chemical residues persist not only in agricultural products but also in soil and water, where they have been associated with harmful effects such as carcinogenicity, endocrine disruption and neurotoxicity (Aktar et al., 2009; Mostafalou and Abdollahi, 2013; Mnif et al., 2011; Amaral et al., 2025). In addition, chemical pesticides have a negative impact on non-target organisms, including soil microbiota. They also promote the emergence of resistant pest populations and contribute to long-term ecological imbalances (Pimentel, 2005; Swaine et al., 2025). Beyond these concerns, insect pests also result in significant economic losses, with annual costs in the United States alone estimated at USD 13.5 billion (Neven et al., 2018). Among the most damaging species in agriculture and stored products are *Ostrinia nubilalis* (*O. nubilalis*), *Spodoptera exigua* (*S. exigua*), *Spodoptera littoralis* (*S. littoralis*), *Grapholita molesta* (*G. molesta*), and *Ephestia kuehniella* (*E. kuehniella*) (Zalucki et al., 2012). These larvae cause extensive economic losses by attacking vital plant structures and stored products. *O. nubilalis* tunnels in corn stems and cobs, reduces yield and leads to secondary fungal infections. *Spodoptera* defoliates a wide range of crops, *G. molesta* bores into fruit tree shoots and fruits, such as peach, plum, and cherry trees, causing dieback and fruit drop, and *E. kuehniella* (the Mediterranean flour moth) infests stored grains and flour causing contamination and quantitative losses (Zalucki et al., 2012). Their feeding habits complicate their control strategies, and the extensive use of chemical insecticides has led to pest resistance. Consequently, there is a critical need for a new generation of effective, sustainable biopesticides, not only to control these pests but also to reduce the use of chemical pesticides (Yuan et al., 2021; Pintilie et al., 2023; Herrero et al., 2016; Neven et al., 2018).

In this context, biological control presents a safer alternative to chemical insecticides. Biopesticides derived from natural sources are effective in controlling pests while limiting negative ecological impacts (Isman, 2006). *Bacillus thuringiensis* (*B. thuringiensis*), the most widely used microbial biocontrol agent, as a vital component of integrated pest management and used as alternative for chemical insecticides (Domínguez-Arrizabalaga et al., 2020; Glare and O'Callaghan, 2000). This Gram-positive, spore-forming soil bacterium is characterized by the production of parasporal crystalline inclusions during sporulation. These inclusions are composed mainly of insecticidal delta-endotoxins (Cry and Cyt proteins), which exhibit high specificity and potent activity against insects from several economically important orders, including Lepidoptera, Coleoptera, and Diptera (Bravo et al., 2011; Jouzani et al., 2017).

Despite of its advantages, the dissemination of large amounts of *B. thuringiensis* spores after field application can lead to a significant ecological concern. Because of their high resistance to environmental stresses, spores can persist in soil, on plant surfaces and in aquatic environments for long periods (De Maagd et al., 2001; Paris et al., 2011). Their prolonged survival may lead to microbial imbalances, including the persistence in non-target habitats resulting food and water contamination (Biggel et al., 2022; Jouzani et al., 2017; Pinnock et al., 1974). These issues highlight the need to develop *B. thuringiensis* strains with reduced sporulation while maintaining their biocontrol efficiency.

Random mutagenesis is a powerful and widely used approach for generating improved biocontrol strains (Ghribi et al., 2004). This strategy involves inducing random genetic alterations through physical or chemical agents to disrupt sporulation pathways without prior knowledge of the underlying genetic targets. Several studies have successfully used random mutagenesis to create asporogenic or oligosporogenic *B. thuringiensis* mutants that maintain high insecticidal activity while exhibiting reduced environmental persistence (Ben Khedher et al., 2011).

Building upon this strategy, we recently studied the feasibility of generating oligosporogenic *B. thuringiensis* BLB1 mutants using a multi-agent mutagenesis approach (nitrous acid, UV, and acridine orange) optimized by the Taguchi method (Sellami et al., 2026). While that study established the high-yield production and toxicity of those mutants, the present work focuses on a new lineage of selected clones generated exclusively through a targeted UV radiation strategy. This specific approach was chosen to minimize the accumulation of non-specific background mutations compared to combined chemical-physical methods, thereby facilitating a more precise identification of the mutations through Whole Genome Sequencing (WGS). In this study, we aimed to characterize and compare two new oligosporogenic mutants of *B. thuringiensis* strain BLB1 at both phenotypic and genomic levels. This approach aims to contribute to the development of safer, environmentally sustainable *B. thuringiensis*-based biological control agents by understanding the specific genetic variations that link a significant reduction in spore dissemination with the maintenance or enhancement of biocontrol efficacy.

## 2 Materials and methods

### 2.1 Bacterial strains and cultivation conditions

The reference strain in this study was *B. thuringiensis* strain BLB1, which was first isolated from a soil sample collected at Tunisia (Jallouli et al., 2014). Then, a new selection of 12 oligosporogenic mutants of *B. thuringiensis* were isolated after several treatments using ultra-violet radiation (UV). The Luria-Bertani (LB) culture medium was used to grow the *B. thuringiensis* strains, while the solid T3 and CCY medium (Stewart et al., 1981; Sellami et al., 2013) were used to produce crystal inclusion bodies during the sporulation growth phase (Sellami et al., 2026). Furthermore, a glucose-based medium explained by (Zouari and Jaoua 1997) was used to enhance the formation of parasporal crystals in flasks with 50 mL of this medium shaken at 200 rpm for 72 h at 30 °C. Additionally, CCY liquid medium was used to induce sporulation of *B. thuringiensis*.

### 2.2 Targeted UV-mutagenesis and phenotypic screening

Unlike the multi-agent approach (nitrous acid, UV, and acridine orange) described by (Sellami et al. 2026), the mutagenesis in this study was carried out using ultraviolet (UV) irradiation (254 nm). This efficient procedure was adopted to facilitate a clearer genomic analysis by reducing the frequency of non-specific background mutations. An isolated colony of the wild-type *B. thuringiensis* strain BLB1 was inoculated into 3 mL of Luria–Bertani (LB) medium and incubated overnight at 200 × g/g and 30 °C. The culture was transferred into 250 mL Erlenmeyer flasks containing 50 mL LB medium, with an initial optical density of 0.15 at 600 nm (OD_600_), then incubated under the same conditions until mid-exponential phase (OD_600_ = 0.5), reached after 4 h (Sellami et al., 2026). After that, cultures were adjusted to OD_600_ = 0.1 and transferred into sterile Petri plates. The Petri plate lid was removed then the plate was exposed to UV light (254 nm) from a 15 W Philips tube positioned 20 cm above the surface. Exposure durations varied from 2 to 60 min. Following irradiation, cells were serially diluted and plated on solid T3 medium. The plates were incubated for 72 h at 30 °C to determine the survival rate (Bouassida et al., 2018).

Survivor colonies were subjected to a two-step screening strategy. Firstly, a morphological screening was performed to select colonies exhibiting an atypical compared to the wild-type strain. The wild-type strain BLB1 typically forms opaque and creamy white colonies, which are characteristics of sporulating *B. thuringiensis* strains producing parasporal crystals. Secondly, the selected colonies were examined using phase-contrast microscopy to determine the ratios of vegetative cells, spores, and parasporal crystal inclusions. Promising mutants, exhibiting low ratios of spores, were subjected to additional rounds of UV mutagenesis to stabilize the oligosporogenic phenotype. Final candidate mutants that exhibited significantly reduced sporulation while maintaining crystal production were selected for further characterization and Whole Genome Sequencing (WGS).

### 2.3 Analysis of growth, sporulation, and crystal protein production

To evaluate bacterial growth and sporulation, post-logarithmic phase cultures were used to determine the number of viable cells and spores by colony forming unit (CFU) counting.

To selectively enumerate spores, samples were subjected to heat treatment at 80 °C for 10 min to eliminate vegetative cells while preserving heat resistant spores, following the established differential thermal resistance between vegetative cells and endospores (Setlow, 2006), then plated on LB agar and incubated at 30 °C for 24 h to assess total sporulation, both before and after heat exposure. Delta-endotoxin levels were measured from solubilized crystal preparations according to (Jamoussi et al. 2013). Briefly, 1 mL of culture was centrifuged at 13,000 rpm for 10 min to collect crystal-spore pellets, which were washed with NaCl and distilled water. The pellets were then incubated in 1 mL of 50 mM NaOH (pH 12.5) for 2 h at 30 °C to solubilize the crystals. After centrifugation at 13,000 rpm for 15 min, the supernatant containing insecticidal crystal proteins was used to quantify delta-endotoxin using the Bradford assay. All experiments were performed in triplicate, with three technical replicates each, and results were averaged and analyzed using Microsoft Excel 2016.

### 2.4 Cry protein analysis

Proteins from a mixture of spore and crystal samples were analyzed using sodium dodecyl sulfate–polyacrylamide gel electrophoresis (SDS-PAGE), as described by (Laemmli 1970). Samples were mixed with Laemmli sample buffer, then heated at 100 °C for 10 min. An equal amount of total protein 10 μg per lane was loaded for each sample, then loaded onto gels (4% stacking layer, 10% resolving layer). Electrophoresis was then carried out at a constant voltage (100 V) until the dye front reached the bottom of the gel. After separation, gels were stained with 0.4% Coomassie Brilliant Blue R-250 and then destained using a solution of 40% methanol, 10% acetic acid, and 50% distilled water until the protein bands were clearly visible. A broad-range protein molecular weight marker was included to estimate the molecular masses of the resolved proteins.

### 2.5 Bioassays of *B. thuringiensis* against lepidopteran pests

The insecticidal activity of the wild-type strain BLB1 and its mutants T3 and T8 was evaluated against five major lepidopteran pests, including *G. molesta, O. nubilalis, S. exigua, S. littoralis*, and *E. kuehniella* to determine whether mutagenesis affected their toxicity.

Bioassays for *G. molesta, O. nubilalis, S. exigua*, and *S. littoralis* were conducted using a surface contamination method on artificial diet as previously described (Hernández-Martínez et al., 2008). Seven doses were used to determine the LC_50_ values of the T3 and T8 mutants, as well as the wild-type strain, against the studied pest. The concentrations used were as follows: for *G. molesta* and *O. nubilalis*, [810, 270, 90, 30, 10, 3.3 and 1.1 ng/cm^2^] and for *S. exigua* and *S. littoralis*, [1,200, 800, 600, 450, 300, 200 and 100 ng/cm^2^]. Each concentration was applied to individual wells containing artificial diet, and 16 larvae were used for each concentration. Wells treated with carbonate buffer alone were used as the negative control.

After air-drying, one neonate larva was placed in each well, and the trays were sealed and incubated for 7 days under controlled temperature, relative humidity, and photoperiod conditions (Toledo et al., 2025). Larval mortality was recorded after 7 days, and median lethal concentrations (LC_50_) were estimated by probit analysis using the POLO-PC program (LeOra Software, Berkeley, CA, USA), enabling comparison of the toxicities of the mutant strains with that of the wild-type BLB1 (Finney, 1971).

For *E. kuehniella*, toxicity tests were carried out on third-instar larvae. Ten larvae were introduced into sterile Petri dishes containing 1 g of flour supplemented with different concentrations of the spore–crystal mixture from the T3 and T8 mutants, as well as the wild-type strain BLB1, and incubated at 27 °C. Flour mixed with water was used as the negative control, while the wild-type strain BLB1 was used as a positive control. Seven different concentrations were used (20, 40, 60, 100, 140, 180 and 200 mg L^−1^) and each concentration was tested in triplicate across three independent experimental runs. The median lethal concentration (LC_50_) values were performed using R software (Venables and Smith, 2004).

### 2.6 Study of kinetics in semi-synthetic media

In this study, growth kinetics and glucose consumption were investigated simultaneously in order to compare the growth dynamics of the mutants with those of the wild-type strain BLB1 in the semi-synthetic medium. Each strain was inoculated into 100 mL of semi-synthetic medium composed of glucose (5 g/L), casein hydrolysate (4.5 g/L), yeast extract (0.5 g/L), (NH_4_)_2_SO_4_ (6 g/L), K_2_HPO_4_ (1.4 g/L), KH_2_PO_4_ (1.4 g/L), MgSO_4_ (0.61 g/L), CaCl_2_ (0.332 g/L), and MnSO_4_ (0.006 g/L). Each strain was inoculated into 100 mL of this medium in 2L Erlenmeyer flasks at an initial optical density of 0.15, before being incubated at 30 °C with shaking at 200 rpm. Samples (1 mL) were collected culture until sporulation. Samples (1 mL) were collected at 0, 2, 4, 6, 8, 10, 12, 14, 16, 18, 20, 22, 24, 28, 32, 36, 48, 56, and 72 h to closely evaluate growth and sporulation kinetics. After that, viable cell and spore counts were determined by CFU enumeration before and after heat treatment at 80 °C for 10 min, as described above. Cells were pelleted by centrifugation at 10,000 rpm for 5 min, after which the glucose concentration in the culture supernatant was determined using the dinitrosalicylic acid (DNS) method (Miller, 1959). All measurements were performed in triplicate for three independent experimental runs.

### 2.7 WGS and genome analysis

Comparative genomic analysis against the wild-type strain BLB1 was conducted to uncover the genetic basis of the sporulation deficiency observed in *B. thuringiensis* mutants T3 and T8. The genome of BLB1 has been previously sequenced, and its data is publicly available under the NCBI BioProject accession number PRJNA1417242. Therefore, T3 and T8 mutants were transferred to African Biotechnology Society to perform the sequencing. DNA was extracted from each mutant and then subjected to purity and concentration control using a QIAxpert spectrophotometer (QIAGEN). The QIAseq FX DNA Library Kit (QIAGEN) was used to construct sequencing libraries according to the manufacturer's specifications. The size distribution and integrity of the final libraries were verified via capillary electrophoresis on a QIAxcel Connect system (QIAGEN), and a Qubit Fluorometer (Thermo Fisher Scientific) was used for the quantification.

The prepared libraries were normalized, pooled, and sequenced using an Illumina MiSeq platform with a MiSeq Reagent Kit v3, yielding paired-end reads (2 × 300 bp). Initial quality assessment of the raw sequencing data was performed using FastQC (Andrews, 2010) to evaluate per-base sequence quality, GC content, and potential adapter contamination. The resulting quality metrics were collected and visualized with MultiQC (Ewels et al., 2016). *De novo* genome assembly for T3 and T8 mutants was carried out with SPAdes v3.15.0 (Bankevich et al., 2012) under default parameters optimized for Illumina paired-end reads. The quality of the draft assemblies was assessed using QUAST (Gurevich et al., 2013), which provided critical metrics including total assembly length, contig count, and N50 values.

Genome annotation of mutants was performed with Prokka (Seemann, 2014) which allows identifying and characterizing coding sequences (CDSs), ribosomal RNAs (rRNAs), transfer RNAs (tRNAs) and other functional elements. This pipeline generated comprehensive, GenBank-compatible annotation files for each strain. After that, a systematic comparative genomic analysis was then executed to identify structural variations and sequence polymorphisms. Snippy tool was used to detect single nucleotide polymorphisms (SNPs) and insertions/deletions (indels) by mapping the sequencing reads of T3 and T8 mutants against the complete BLB1 reference genome, and the tool Snippy-Core was used to combine the multiple outputs into a core SNP alignment (Seemann, 2015).

The complete genome of *B. thuringiensis* mutants was visualized using Proksee. This tool allows a linear and circular visualization of the bacterial genome, where the quality of the sequences can be observed in a graphical format (Grant et al., 2023).

The objective of this analysis was to correlate specific genetic alterations with the previously observed reduction in sporulation capacity.

### 2.8 Statistical analysis

All statistical analyses and graphical representations were performed using R software (version 4.3.2). Experimental data were analyzed using analysis of variance (ANOVA) and multiple comparisons between strains were conducted using Dunnett's *post hoc* test, with the wild-type BLB1 strain used as the reference. Additionally, the correlation between UV exposure duration and mortality rate was assessed using Pearson's correlation coefficient. Differences were considered statistically significant at *p* < 0.05. Graphs were generated using customized R scripts to ensure reproducibility and consistency of data visualization.

## 3 Results

### 3.1 Iterative UV-mutagenesis and screening strategy for the isolation of low-sporulating *B. thuringiensis* mutants

**Figure 1:**
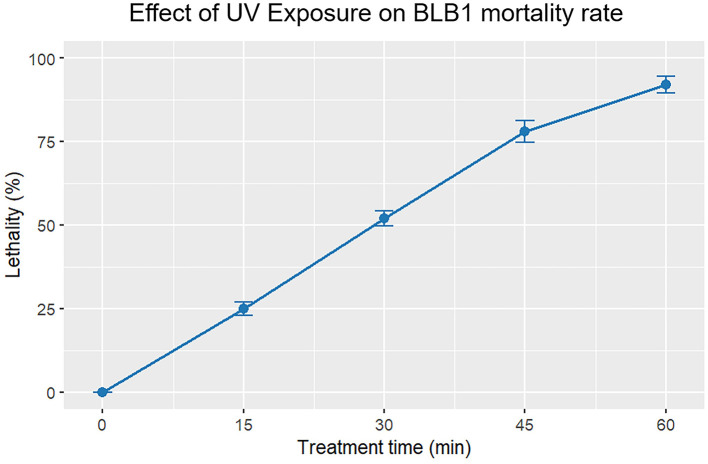
Effect of UV exposure duration on the mean mortality rate of *B. thuringiensis* BLB1.

In the current study, we evaluated the susceptibility of wild-type *B. thuringiensis* BLB1 vegetative cells to ultraviolet (UV) irradiation by monitoring colony-forming units (CFUs). As shown in Figure 1, a direct relationship between dose and response was observed, such that increased exposure time was associated with a progressive decrease in cell survival (Pearson's *r* = 0.984, *p* = 0.0025). Notably, after 60 min of UV treatment, 87% lethality was obtained, confirming the high mutagenic potential of the UV exposure at 254 nm. In fact, based on the new mutagenesis strategy adopted in this work, independent mutagenesis runs were used to maximize genetic variability while targeting a reduction in sporulation capacity. We obtained 150 surviving mutant colonies, and the initial morphological screening of the UV survivors showed no significant differences compared to the wild-type BLB1. Consequently, a systematic microscopic screening was performed using phase-contrast microscopy to assess the relative proportions of vegetative cells, spores, and parasporal crystalline inclusions. Based on these observations, 57 mutants demonstrating interesting decrease in spore number compared to vegetative were selected for further evaluation in 1 L Erlenmeyer flask cultures. From this initial screening, two promising UV-derived mutants, UV1 and UV4, were identified due to their reduced sporulation capacities (5 × 10^7^ ± 1.42 × 10^5^ spores/mL and 6 × 10^7^ ± 1.42 × 10^5^ spores/mL, respectively compared to 2.9 × 10^8^ ± 1.3 × 10^7^ spores/mL for the wild strain BLB1). To further enhance genetic variability and consolidate the oligosporogenic characteristic, these two candidates were subjected to a second round of UV mutagenesis. The UV1 treatment resulted in complete lethality, whereas the second UV exposure of UV4 produced a viable population of 50 mutants. All these mutants were the subject of microscopic observation and cultivation in 1L flask. Among all survivors, the mutant UV4.1 that showed the most interesting decrease in spores' number (36 × 10^6^ spores/ml), compared to 2.9 × 10^8^ spores/mL for the wild strain BLB1, was selected for the third round of UV mutagenesis run in order to maximize the mutational efficiency. Thirty surviving mutants were obtained from this final UV round and then subjected to further examination. Several mutants showed notable morphological changes, including a translucid aspect. Subsequently, a microscopic examination was carried out to evaluate spores ratios, followed by cell culture in 1L flasks, which allowed us to identify mutants with promising decrease in the sporulation capacity. Finally, 12 oligosporogenic mutants with significant decrease in sporulation were isolated using this mutagenesis strategy. These strains might hold potential for further investigation to better understand sporulation regulation and enhance the bioinsecticidal performance.

### 3.2 Evaluation of sporulation response in CCY solid medium

**Figure 2:**
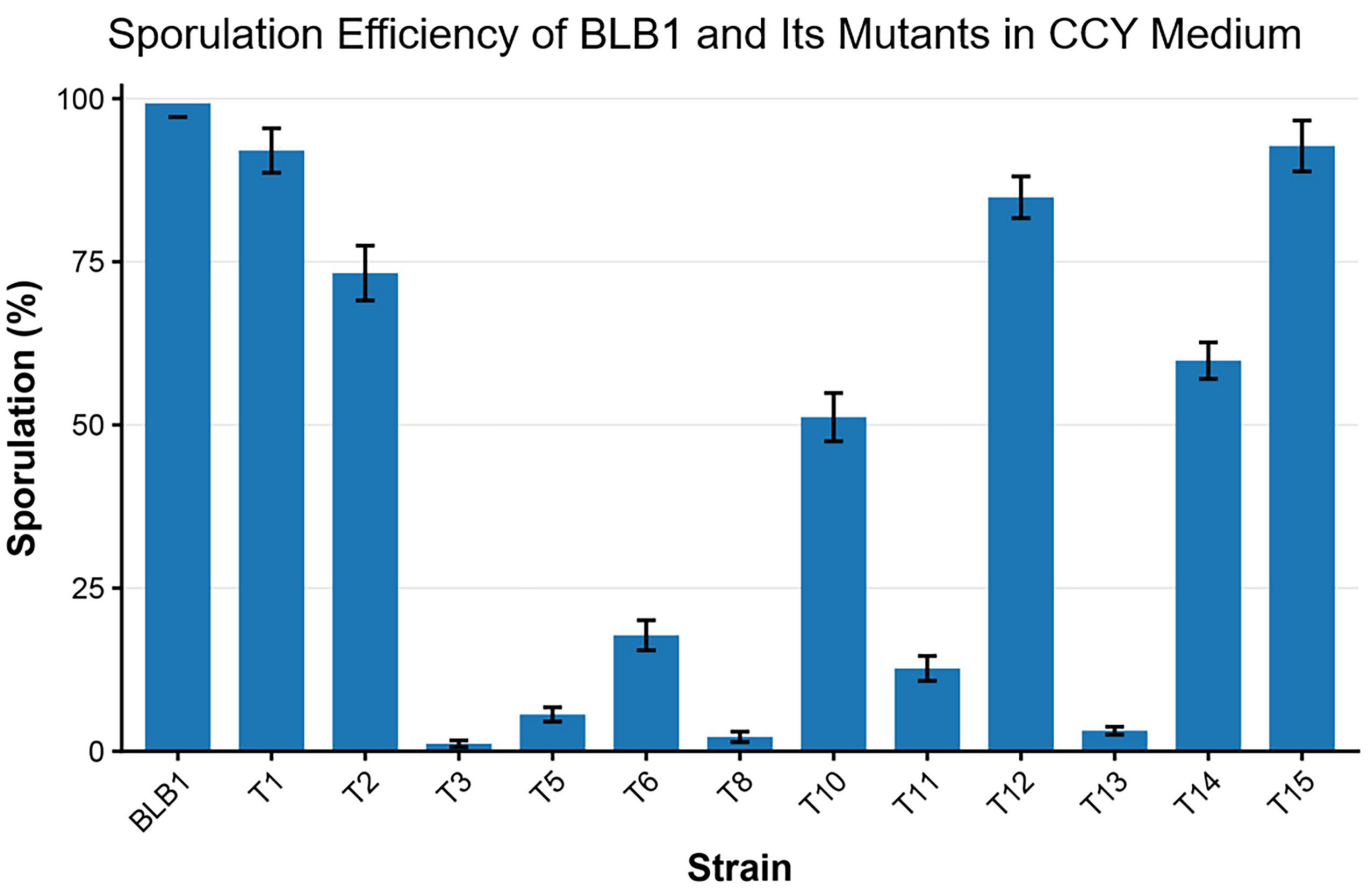
Graph representing sporulation efficiency of *B. thuringiensis* BLB1 and its mutants in CCY media, expressed as percentage of spores relative to total cells by microscopy-based estimation.

Sporulation ability was evaluated by incubating the selected mutants on CCY plates for 48 h to induce sporulation, followed by observation under phase-contrast microscopy. Sporulation efficiency was calculated as the proportion of spores relative to the total cell population observed. The results allowed a clear comparison of the sporulation levels between the wild-type strain BLB1 and the different mutants (Figure 2). As expected, the wild-type strain had the highest level of sporulation (close to 100 %) while a wide range of sporulation rates was observed among the different mutants. Mutants T3, T5, T8, T11, and T13 showed a severe defect in sporulation, as indicated by low spore levels compared to the vegetative cells formed, suggesting possible perturbations in genes responsible for spore formation. Other mutants such as T6, T10, and T14 showed a moderate level of sporulation ability while T1, T2, T12, and T15 mutants exhibited high sporulation rates that were slightly lower than those of the wild-type strain. Based on these results, these mutants confirmed their oligosporogenic characteristic which is reflected by significantly reduced spore formation under CCY-inducing conditions. This difference in sporulation response demonstrates the ability of the tested mutants to produce fewer spores even when exposed to an inducing sporulation medium.

### 3.3 Impact of random mutagenesis on sporulation and delta-endotoxin production in glucose-based medium

In order to evaluate the effect of random mutagenesis on delta-endotoxin production in these 12 selected mutants, we assessed their delta-endotoxin synthesis in a glucose-based medium. Table 1 shows the bioperformance of different selected mutants, including their total cell counts, viable spore presence, and delta-endotoxin yield. All mutants showed different levels of sporulation and protein production compared to the wild-type strain *B. thuringiensis* BLB1 (Table 1).

**Table 1:** Bioperformance of the preselected *B. thuringiensis* mutants generated using three rounds of UV mutagenesis in glucose-based media: data are presented as mean ± standard deviation (SD**)** of three independent replicates.

Strain	Total cells ±SD (CFU/ml)	Spores ±SD (CFU/ml)	Delta-endotoxins ±SD (mg/ml)
**BLB1**	5.95 × 10^8^ ± 1.18 × 10^7^	3.10 × 10^8^ ± 3.75 × 10^7^	2.00 ± 0.14
**T1**	9.20 × 10^7^ ± 0.97 × 10^7^	6.07 × 10^7^ ± 9.14 × 10^6^	0.94 ± 0.09
**T2**	3.25 × 10^8^ ± 3.74 × 10^7^	6.00 × 10^7^ ± 4.58 × 10^6^	1.43 ± 0.12
**T3**	2.01 × 10^8^ ± 4.25 × 10^7^	5.20 × 10^7^ ± 6.90 × 10^6^	1.52 ± 0.20
**T5**	3.75 × 10^8^ ± 6.17 × 10^7^	1.72 × 10^7^ ± 1.31 × 10^6^	0.68 ± 0.21
**T6**	3.36 × 10^8^ ± 4.04 × 10^7^	1.49 × 10^8^ ± 7.69 × 10^7^	1.68 ± 0.27
**T8**	1.23 × 10^8^ ± 5.69 × 10^7^	1.32 × 10^7^ ± 2.07 × 10^6^	1.01 ± 0.09
**T10**	4.56 × 10^8^ ± 8.47 × 10^7^	4.38 × 10^7^ ± 5.57 × 10^6^	1.07 ± 0.17
**T11**	6.09 × 10^8^ ± 7.15 × 10^7^	1.33 × 10^6^ ± 1.00 × 10^5^	0.83 ± 0.11
**T12**	3.19 × 10^8^ ± 0.99 × 10^7^	3.62 × 10^7^ ± 4.76 × 10^6^	1.55 ± 0.21
**T13**	8.88 × 10^7^ ± 5.74 × 10^7^	5.00 × 10^5^ ± 8.51 × 10^4^	0.51 ± 0.08
**T14**	7.71 × 10^8^ ± 6.00 × 10^7^	5.92 × 10^7^ ± 7.42 × 10^6^	1.09 ± 0.13
**T15**	1.71 × 10^8^ ± 2.57 × 10^7^	2.85 × 10^7^ ± 1.28 × 10^6^	0.73 ± 0.09

The wild type BLB1 showed the highest spore count and protein level (3.10 × 10^8^ ± 3.75 × 10^7^ spores/mL and 2.00 ± 0.14 mg/mL) while several mutants showed a significant decrease in spore formation, indicating their oligosporogenic phenotype. For example, T3 and T8, exhibited only 5.20 × 10^7^ ± 6.90 × 10^7^ and 1.32 × 10^7^ ± 2.07 × 10^6^ spores/mL, respectively, indicating their defect in sporulation compared to the wild-type (*p*-value < 0.01 using the Dunnett's *post hoc* test). Interestingly, both mutants maintained relatively high levels of delta-endotoxin production (1.52 ± 0.20 and 1.01 ± 0.09 mg/mL, respectively) which were close to the wild-type.

Moreover, T11 and T13 showed a significant decrease in spore production (1.33 × 10^6^ ± 1.00 × 10^5^ and 5.00 × 10^5^ ± 8.51 × 10^4^ spores/mL, respectively) compared to the wild-type BLB1 (3.10 × 10^8^ ± 3.75 × 10^7^ spores/mL), which was associated with reduced production of delta-endotoxins, with 0.83 ± 0.11 mg/mL for T11 and 0.51 ± 0.08 mg/mL for T13. Additionally, the T12 mutant showed a balanced profile explained by a moderate sporulation (3.62 × 10^7^ ± 4.76 × 10^6^ spores/mL) and a relatively high toxin level (1.55 ± 0.21 mg/mL).

Mutants T3 and T8 were selected for further analysis, as both mutants produced significantly fewer spores (*p* < 0.01) while maintaining protein levels comparable to those of the wild-type BLB1 strain. The stability of the sporulation deficiency was confirmed after 20 successive generations, during which both mutants consistently exhibited reduced spore production compared to the wild-type strain.

### 3.4 Analysis of growth, sporulation, and delta-endotoxin production in CCY liquid medium

The selected mutants T3 and T8 were further characterized in CCY liquid medium, a sporulation-inducing medium commonly used to evaluate *B. thuringiensis* bioperformance. Growth, spore production, and protein synthesis were evaluated and compared with those of the wild-type BLB1 strain. Cultivation in CCY medium allowed assessment of whether the reduced sporulation phenotype observed in the mutants was maintained under conditions favorable for sporulation, while simultaneously evaluating their capacity to support Cry protein production (Table 2).

**Table 2:** Comparative bioperformance of *B. thuringiensis* BLB1 and its mutants T3 and T8 in CCY liquid medium in terms of growth, sporulation, and protein synthesis: Each point represents the mean of three independent replicates ± SD.

Strain	Total cells ±SD (CFU/ml)	Spores ±SD (CFU/ml)	Delta-endotoxins ±SD (mg/ml)
**BLB1**	1.40 × 10^9^ ± 1.7 × 10^9^	1.03 × 10^9^ ± 2.3 × 10^9^	3.84 ± 0.19
**T3**	1.32 × 10^9^ ± 3.5 × 10^8^	5.42 × 10^7^ ± 1 × 10^7^	1.75 ± 0.16
**T8**	1.03 × 10^9^ ± 2.4 × 10^9^	3.40 × 10^7^ ± 9.4 × 10^5^	1.54 ± 0.06

A highly significant reduction in spore production for both mutant strains compared to BLB1 was observed. Spore counts were reduced by approximately 19-fold in T3 and 30-fold in T8 (*p* value < 0.01), confirming the strong oligosporogenic phenotype of the selected mutants (Table 2). Additionally, delta-endotoxin concentrations were decreased to 1.75 ± 0.16 mg/mL in T3 and 1.54 ± 0.06 mg/mL in T8 compared to 3.84 ± 0.19 mg/mL for the wild-type for both mutants (*p* value < 0.001).

**Figure 3:**
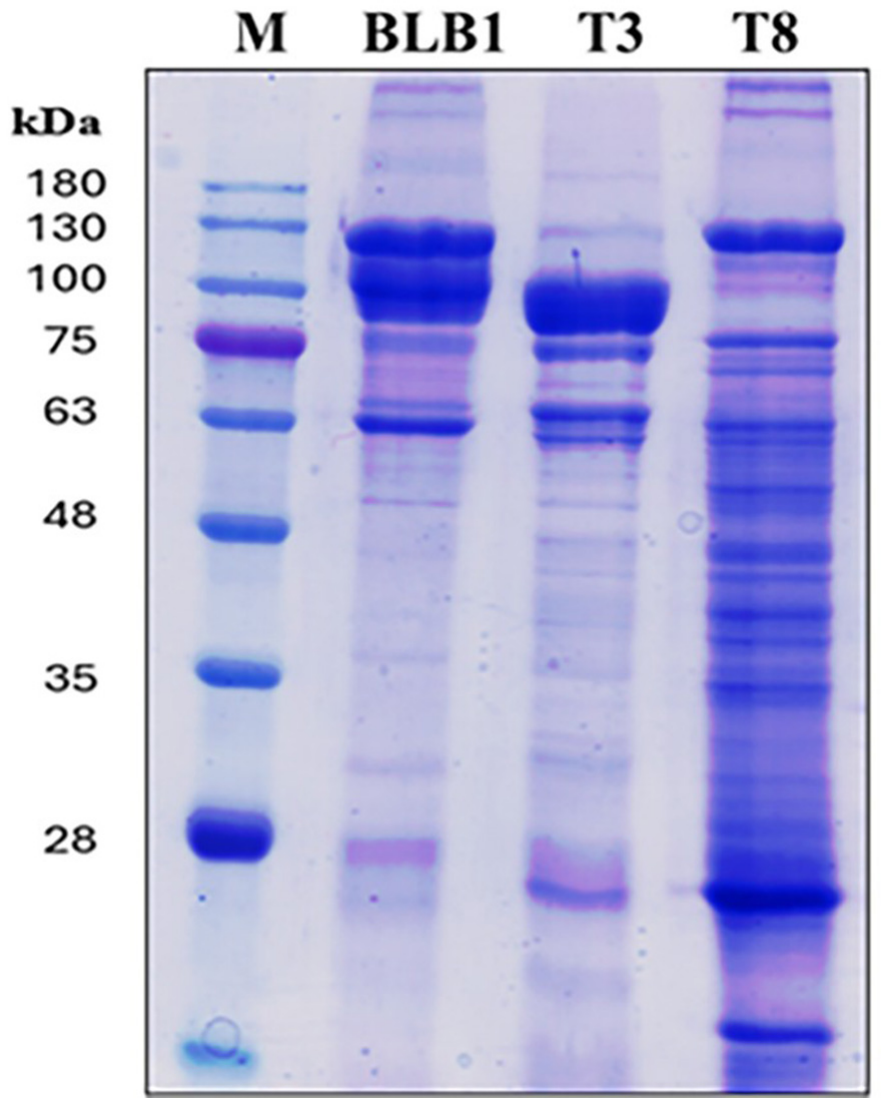
Comparative SDS-PAGE of solubilized proteins from *B. thuringiensis* BLB1, T3, and T8 strains (M: Blue Star marker).

To assess Cry protein expression in the mutants, equal amounts of protein were extracted and analyzed by SDS-PAGE. As shown in Figure 3, the wild-type strain BLB1 displayed prominent bands at ~130 kDa, 100 kDa, and 63 kDa, corresponding to Cry1 and Cry2 protoxins (or activated Cry1 toxin), indicating important toxin production and a high total protein concentration (3.84 ± 0.19 mg/mL). In contrast, the T3 and T8 mutants displayed different protein profiles and altered protein profiles, consistent with their lower delta-endotoxin levels (1.75 ± 0.16 mg/mL for T3 and 1.54 ± 0.06 mg/mL for T8). Notably, the 130 kDa band was absent in T3, whereas the 100 kDa band was absent in T8, indicating distinct alterations in toxin composition between the two mutants rather than a reduction in Cry protein expression. Despite these reductions, Cry protein production remained detectable in the mutants and comparable to the wild type. These results indicate that while the mutations primarily reduce the accumulation of Cry proteins, they do not alter the composition or integrity of the toxins. Thus, despite reduced sporulation and protein yield, both T3 and T8 retain the main Cry protein profile of the wild-type, with minor differences in band intensity and the absence of some low-abundance proteins. Altogether, statistical analyses confirm that UV mutagenesis led to a significant reduction in sporulation and protein accumulation without affecting Cry protein expression.

### 3.5 Bioassay of *B. thuringiensis* against lepidopteran pests

**Figure 4:**
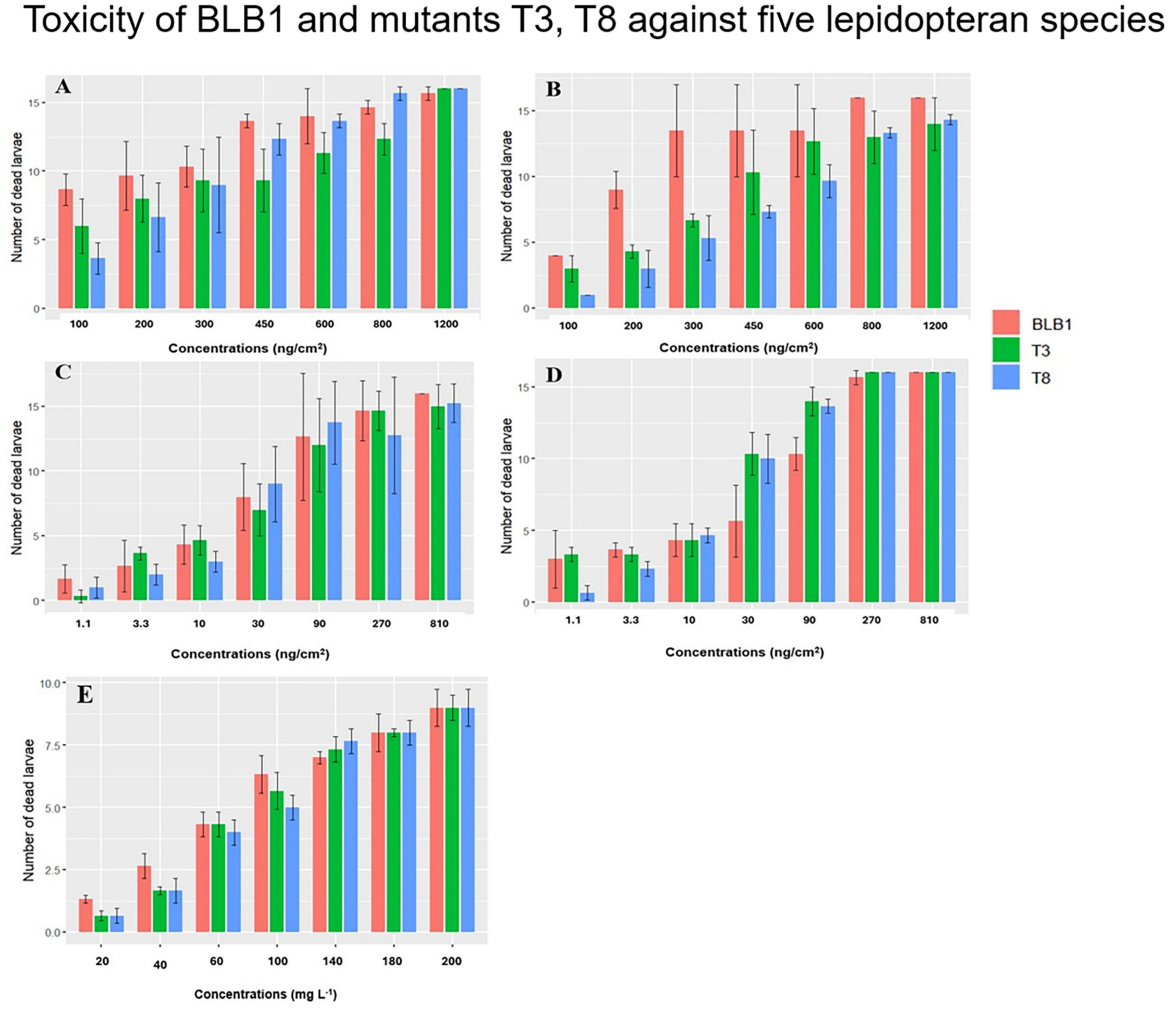
Toxicity of *B. thuringiensis* BLB1 strain and its oligosporogenic mutants T3 and T8 strains against five lepidopteran species: **(A)** *Spodoptera exigua*, **(B)** *Spodoptera littoralis*, **(C)** *Ostrinia nubilalis*, **(D)** *Grapholita molesta* and **(E)** *Ephestia kuehniella*.

To determine whether the observed reductions in sporulation and crystal protein levels affected the biological potential of the selected mutants, we conducted comparative bioassays against five major lepidopteran pests. The insecticidal activity of the wild-type strain BLB1 and the mutants T3 and T8 was evaluated against *G. molesta, O. nubilalis, S. exigua, S. littoralis* and *E. kuehniella* using dose response bioassays. For all species, larval mortality increased with increasing concentrations, allowing reliable estimation of LC_50_ values after seven days of exposure (Figure 4 and Table 3).

**Table 3:** Parameters of the insecticidal activity of *B. thuringiensis* BLB1 and its mutants T3 and T8 against *Spodoptera exigua, Spodoptera littoralis, Ostrinia nubilalis*, and *Grapholita molesta*.

Pest species	Strain	LC_50_ (ng/cm^2^)	Fiducial limits (95%)	Slope ±SD	χ2 (*df*)	*P* value
*Spodoptera exigua*	BLB1	131	82 to 175	1.71 ± 0.26	18 (19)	0.95
	T3	205	130 to 274	1.52 ± 0.23	25 (19)	0.32
	T8	258	209 to 302	5.11 ± 1.44	21 (19)	0.67
*Spodoptera littoralis*	BLB1	180	101 to 248	2.82 ± 0.38	19 (11)	0.12
	T3	369	299 to 438	2.62 ± 0.34	22 (19)	0.56
	T8	432	379 to 490	2.89 ± 0.30	13 (19)	0.32
*Ostrinia nubilalis*	BLB1	18	12 to 27	1.22 ± 0.24	24 (17)	0.24
	T3	32	19 to 52	1.20 ± 0.11	32 (19)	0.06
	T8	26	19 to 35	1.66 ± 0.14	15 (20)	0.24
*Grapholita molesta*	BLB1	25	15 to 40	1.15 ± 0.12	25 (15)	0.45
	T3	17	10 to 26	1.46 ± 0.15	31 (19)	0.08
	T8	17	13 to 23	1.62 ± 0.14	9 (19)	0.05

Goodness of fit was evaluated using a chi-square (χ^2^) test performed with the POLO software program, and *P*-values were obtained from a two-tailed distribution. SD, standard deviation; df, degrees of freedom.

Bioassays against *G. molesta* and *O. nubilalis* revealed that LC_50_ values of both mutants were in the same order of magnitude as those of the wild-type, indicating that mutagenesis did not markedly affect toxicity toward these species. Interestingly, both mutants showed lower LC_50_ values against *G. molesta* (17 ng/cm^2^ for both mutants T3 and T8) compared with the wild-type strain (25 ng/cm^2^), highlighting their potential interest for the control of this pest (Table 3). Additionally, T3 exhibited measurable toxicity against *O. nubilalis*, with an LC_50_ of 32 ng/cm^2^, although this value indicates a lower toxicity compared to the wild-type strain BLB1 (18 ng/cm^2^). T8 showed LC_50_ of 26 ng/cm^2^, indicating a toxicity level closer to that of the wild-type and higher than that of T3. Moreover, the 95% fiducial limits indicate that the mutants' toxicity is generally comparable to that of the wild-type.

In contrast, BLB1 showed LC_50_ values of 131 ng/cm^2^ and 180 ng/cm^2^ against *S. exigua* and *S. littoralis*, respectively, whereas T3 displayed LC_50_ values of 205 ng/cm^2^ and 369 ng/cm^2^, and T8 showed the highest LC_50_ values of 258 ng/cm^2^ and 432 ng/cm^2^ (Table 3). These results suggest that the mutations, particularly in T8, may have a stronger impact on toxin activity against this species, highlighting the strain and species-specific effects of genetic modifications on *B. thuringiensis* insecticidal potency (Table 3).

Additionally, bioassays performed *E. kuehniella* showed that BLB1 exhibited the lowest LC_50_ value (72.39 ± 36 mg L^−1^), while slightly higher LC_50_ values were obtained for the T3 and T8 mutants (78.33 ± 31.09 mg L^−1^ and 87.29 ± 19.15 mg L^−1^, respectively), suggesting that the wild-type strain and its oligosporogenic mutants display similar levels of toxicity” (Figure 4).

### 3.6 Delayed sporulation and extended glucose consumption in semi-synthetic media

**Figure 5:**
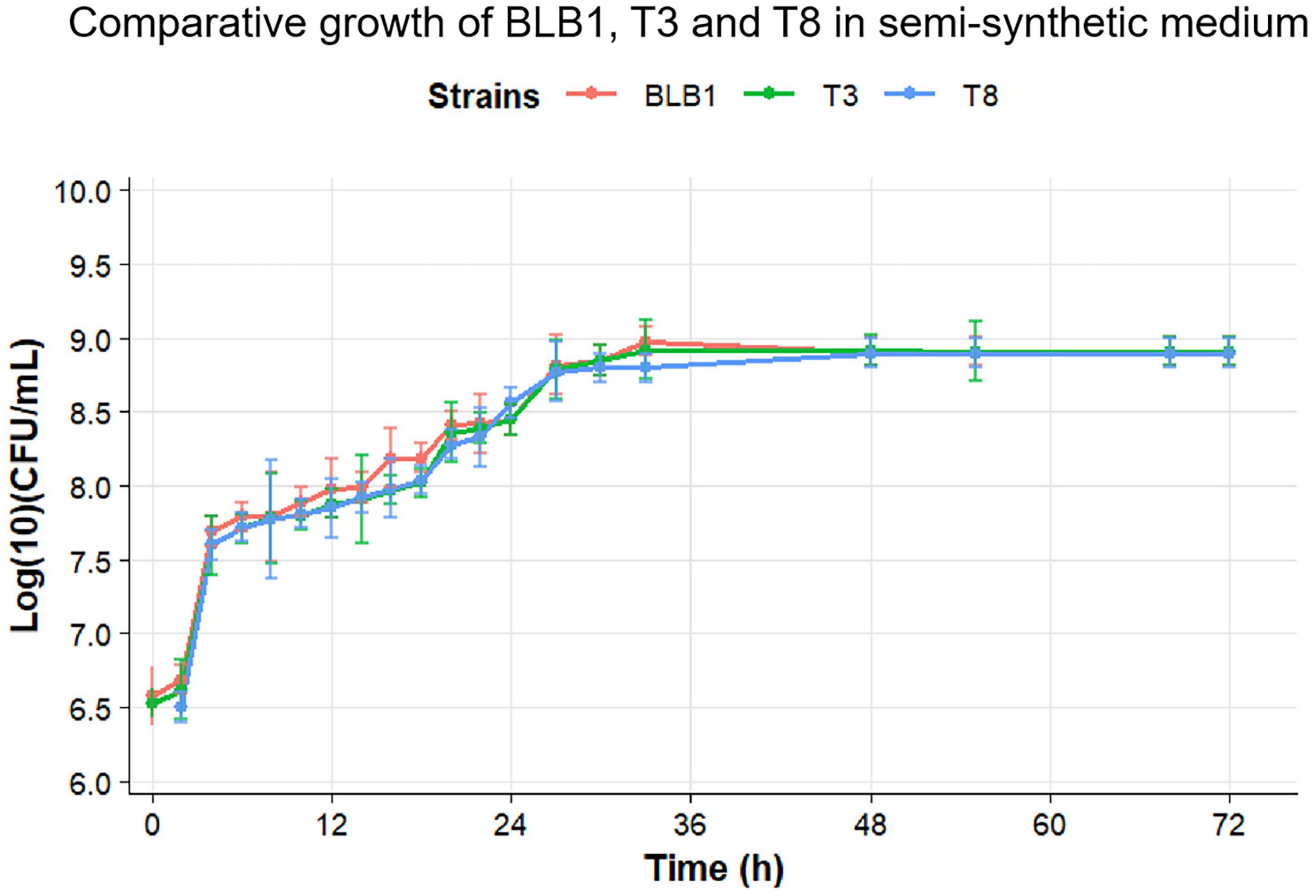
Comparative growth dynamics of *B. thuringiensis* BLB1 and its oligosporogenic mutants T3 and T8 in semi-synthetic medium: Each point represents the total cell count corresponding to the mean of three independent replicates.

**Figure 6:**
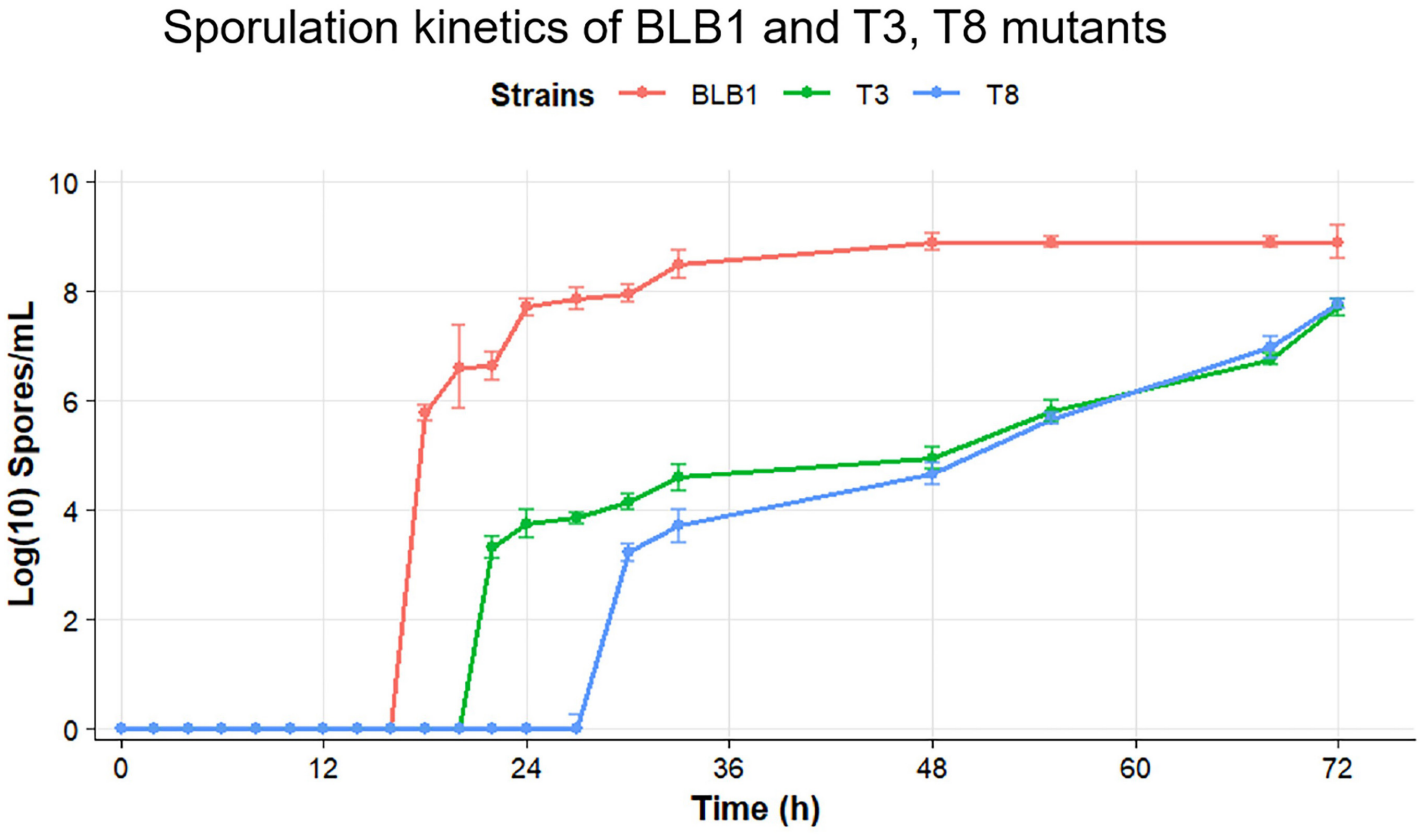
Kinetics of sporulation of *B. thuringiensis* BLB1 and its oligosporogenic mutants T3 and T8 in semi synthetic media: Each data point corresponds to the mean spores count obtained from three independent biological replicates.

To understand the behavior of the T3 and T8 mutants, a kinetic study was conducted in semi-synthetic medium where samples were collected over 72 h to estimate the vegetative growth, sporulation, and glucose consumption. The obtained results allowed a clear distinction between the two mutants as well as the wild-type strain. Similarly to BLB1, the T3 mutant demonstrated robust vegetative growth during the initial hours of cultivation, reaching comparable optical density values (Figure 5). However, despite its sustained growth, T3 exhibited a delayed and less efficient sporulation process. BLB1 started the sporulation in about 20 h, reaching a maximum of log_10_ = 8.5 spores/mL in 48 hours. In contrast, T3 initiated the sporulation later (around 22h) and a significant reduction in spore production was observed, reaching a maximum of log_10_ =6.77 spores/mL at 72 h, indicating an oligosporogenic phenotype. Furthermore, glucose was consumed more rapidly in BLB1, with complete consumption by 16 h, while T3 showed a slow glucose consumption which was consistent with the prolonged vegetative phase (Figure 6).

**Figure 7:**
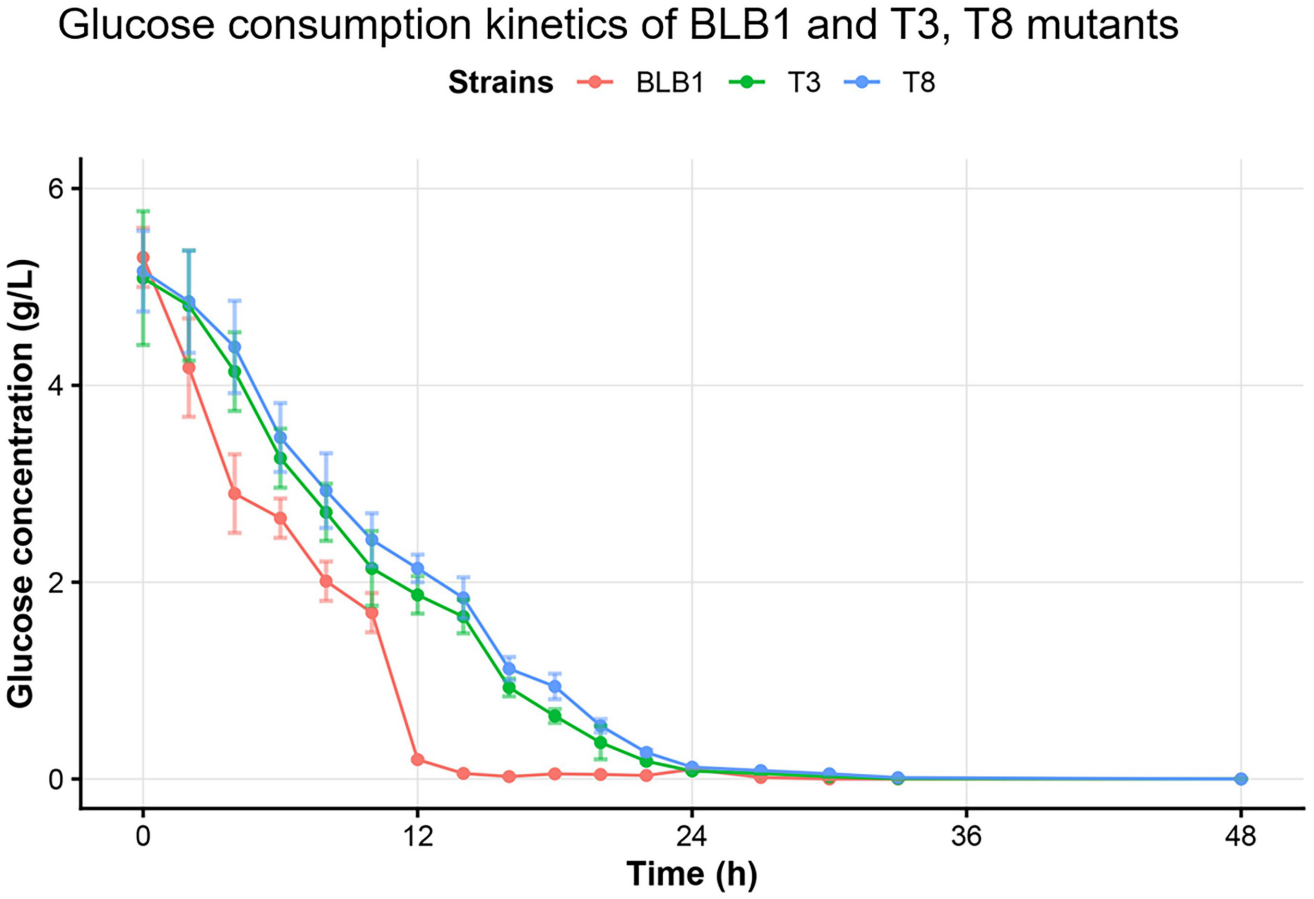
Kinetics of glucose consumption of *B. thuringiensis* BLB1 and its oligosporogenic mutants T3 and T8 in semi synthetic medium. Glucose concentration shows its value in the medium at the growing time indicated.

This delay in sporulation kinetics is reflected in glucose consumption. T3 consumed glucose at a lower rate than BLB1, particularly during its exponential phase. For instance, during the first 6 h, T3 metabolized 1.83 g/L of glucose (from 5.09 to 3.26 g/L), while BLB1 consumed 2.65 g/L (from 5.30 to 2.65 g/L). By 12 h, BLB1 had used more than 96% of the available glucose, while T3 had only reduced glucose to 1.87 g/L, reflecting a slower level of consumption. At 20 h of culture, BLB1 had nearly depleted its glucose reserves (0.046 g/L), whereas T3 still maintained 0.37 g/L and reached complete depletion around 33 h (Figure 7).

These findings suggest that although T3 can maintain vegetative growth, its metabolic activity, reflected by glucose consumption and delayed sporulation, is reduced compared to the wild-type strain.

Likewise, the T8 mutant also showed lower vegetative growth than the wild-type BLB1 during the early phases of cell culture (Figure 5). However, it exhibited a significant delay in onset of sporulation which was associated with a low spore production. While BLB1 began the sporulation at around 20 h and reached a maximum spore count of log_10_ = 8.50 spores/mL by 48 h, T8 did not initiate sporulation until 33 h and achieved its maximum of log_10_ = 6.97 spores/mL at 72 hours (Figure 6). Moreover, noticeably slower glucose consumption was observed in T8 compared to BLB1. After 6 h, T8 had consumed approximately 1.69 g/L of glucose (from 5.16 to 3.47 g/L), while BLB1 had already used 2.65 g/L (from 5.30 to 2.65 g/L). After 12 h, T8 consumed only 2.14 g/L while BLB1 had used over 96% of the available glucose (reduced to 0.197 g/L). Interestingly, complete glucose consumption was observed by 20 h for the wild strain BLB1, whereas T8 still had measurable levels of glucose (0.54 g/L) at the same time point and did not fully consume its carbon source until approximately 33 h (Figure 7). This delay in glucose consumption was consistent with the lower sporulation levels indicating altered metabolic pathway in T8 mutant. When combined, these results reinforce the oligosporogenic profile of T3 and T8, reflecting their distinction from the wild type BLB1 in terms of sporulation and glucose consumption. This decreased metabolic activity, associated with the delayed and limited sporulation, supports the classification of T3 and T8 as oligosporogenic mutants.

### 3.7 Genome analysis of *B. thuringiensis* BLB1 mutants

In order to associate phenotypic alterations detected in the T3 and T8 mutants with their genetic basis, WGS was performed using paired-end Illumina technology (2 × 150 bp). After quality control and trimming, high-quality reads were assembled *de novo* using Spades resulting in assemblies with different characteristics. The T3 genome was assembled into 37 contigs with a total length of 493788 bp (N50: 36.5 Kbp), while the T8 genome consisted of 8169 contigs totaling 4295252 bp (N50: 0.9 Kbp). These findings suggest that UV treatment has extremely affected genome completeness and may have resulted resulted in severe DNA breaks at the genome level, which is reflected in the high complexity of the assembly.

Despite these assembly challenges, Prokka annotation predicted 546 coding sequences (CDSs), 3 rRNA genes, and 1 tRNA gene for T3 mutant while T8 demonstrated 3712 CDSs, 3 rRNA genes, and 2 tRNA genes. Additionally, two repeated regions were detected in T3 mutants and 15 in T8, suggesting the presence of structural rearrangements or sequence duplications resulting from the mutagenic treatment (Table 4). These findings, particularly for the T3 mutant, reflect the incomplete and highly fragmented nature of these assemblies, rather than biological differences. Which is consistent with the observed low sequencing coverage and assembly metrics for T3 mutant, indicating that many genes may have been lost during annotation.

**Table 4:** Summary of WGS and annotation results of *B. thuringiensis* mutants T3 and T8.

Genomic features of mutants	T3	T8
Number of contigs	37	8,169
Total assembly length (bp)	493,787	4,295,252
N50 (Kbp)	36.5	0.9
Coding sequences (CDS)	546	3,712
rRNA	3	3
tRNA	1	2
Repeated regions	2	15

**Figure 8:**
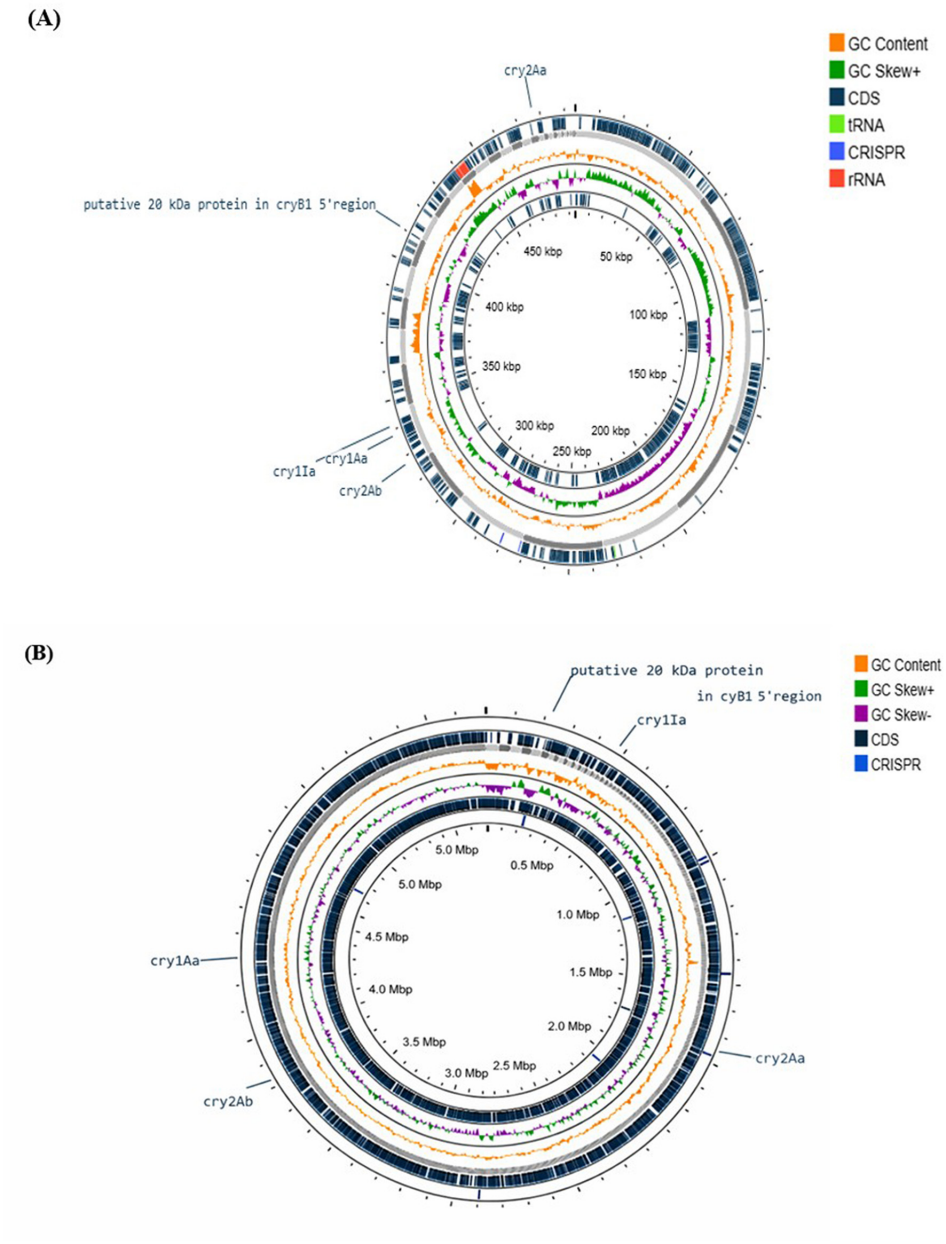
Circular genome maps of *B. thuringiensis* oligosporogenic mutants generated using the Proksee tool: **(A)**: T3 mutant genome and **(B)**: T8 mutant genome.

The circular genome map presented in Figure 8 illustrates the major genomic features distributed across both DNA strands. The legend represent the predicted coding sequences (CDS) on the forward and reverse strands, colored in blue, the orange plot denotes the GC content, while the green and purple plots correspond to the GC skew (+ and –), respectively, reflecting compositional asymmetry along the genome.

Functional genomic elements like tRNA genes, rRNA operons, CRISPR arrays and intact prophage regions are indicated by distinct colors. Additionally, several key insecticidal crystal (Cry) toxin genes were annotated, including *cry1Aa, cry1Ia, cry2Ab*, and *cry2Aa* as well as a putative 20 kDa protein located in the *cryB1* 5′ region. These loci are characteristic of *B. thuringiensis* subspecies and contribute to its insecticidal activity (Figure 8).

These genomic features support the preservation of typical *B. thuringiensis* genomic organization; with *cry* genes localized in distinct clusters often associated with plasmid regions or mobile genetic elements. In both T3 and T8 mutants, multiple plasmids were identified, showing the typical plasmid complexity characteristic of *B. thuringiensis*. Unlike the BLB1 wild-type strain, which contains 11 plasmids ranging from 2 to 457 kb; 8 plasmids were identified for T3 and 11 for T8 (Table 5). T3 revealed 8 plasmids ranging from approximately 6.5 kb to 278 kb, and T8 contained 12 plasmids, with lengths ranging between 2.1 kb and 317 kb, but with a slightly better continuity than T3 (Table 5). These plasmids contained different elements including CDS for pesticidal toxins, insertion sequences and hypothetical proteins.

**Table 5:** Sequence features and accession numbers of replicons from T3 and T8 mutants of *B. thuringiensis* strain BLB1.

Mutant	Replicon	Length (bp)	CDS	Cry genes
T3	Chromosome	46,734	55	—
	Plasmid_AA656	7,413	9	—
	Plasmid_AA661	6,535	9	—
	Plasmid_AA783	278,045	264	*cry2Ab; cry1Aa; cry1Ia;* putative 20KDa *cryB1, cry2Aa*
	Plasmid_AB999	8,334	11	
	Plasmid_AD034	76,703	79	
	Plasmid_AD181	20,210	34	
	Plasmid_AD536	47,697	76	
	Plasmid_AF086	7,413	4	
T8	chromosome	3,683,022	3,071	*cry2Ab; cry1Aa; cry1Ia;* putative 20KDa *cryB1; cry2Aa*
	Plasmid_AA656	8,993	12	
	Plasmid_AA661	2,214	3	
	Plasmid_AA783	317,370	299	
	Plasmid_AA951	59,622	69	
	Plasmid_AA979	49,865	60	
	Plasmid_AB999	8,334	11	
	Plasmid_AD034	65,588	88	
	Plasmid_AD181	14,924	24	
	Plasmid_AD370	14,893	27	
	Plasmid_AD536	49,350	73	
	Plasmid_AF086	2,117	3	
	Plasmid_AG540	18,960	26	

BLAST analysis of the AA783 plasmid from both mutants against the pBtoxis plasmid of the wild-type BLB1 strain revealed a very high level of sequence conservation, with approximately 99–100% nucleotide identity. Among these, this mutant's plasmid carried gene clusters encoding several insecticidal proteins, including *cry2Ab, cry1Aa, cry1Ia*, the putative 20 kDa *cryB1*, and *cry2Aa*. These toxin-encoding genes were clearly annotated and visualized in the circular plasmid maps (Figure 8). Notably, T3 and T8 mutants showed a similar plasmid organization compared to the well-characterized pBLB1_317 plasmid of the *B. thuringiensis* BLB1 strain, including all characterized *cry* genes such as *cry1Aa, cry1Ia, cry2Aa, cry2Ab* and the 5′ region of *cryB1*, indicating a higher level of plasmid conservation.

In addition to the *cry* toxin genes, other virulence-related factors were identified on the AA783 plasmid. Both mutants contained four copies of a chitinase-encoding gene, indicating that this feature is conserved despite mutagenesis. In contrast, a metalloprotease-encoding gene was detected only in the T8 mutant, suggesting a strain-specific plasmid modification.

Moreover, to further characterize the effect of mutagenesis on selected mutants, the Snippy tool was used to detect mutations in the genomes of the two studied mutants by mapping them to the reference genome of the wild strain BLB1, which revealed distinct mutation profiles with significant functional implications. In fact, genome analysis of the T3 mutant revealed seven single-nucleotide polymorphisms (SNPs) with high confidence scores (from 255.39 to 2052.52) (Table 6A). Three of these SNPs (A → C, G → A, and C → T) were clustered within a gene encoding a hypothetical protein (PJW01_27745), suggesting that this region may be functionally inhibited despite its unknown role. A further high-quality mutation (T → A; score 995.65) was identified in an enterotoxin-encoding gene (PJW01_09655), potentially affecting virulence. Moreover, two additional SNPs were detected in a phage tail protein pseudogene (PJW01_29655), likely reflecting the inactivation of a mobile genetic element. By comparison, the T8 mutant carried only four SNPs, supported by moderate but reliable quality scores (266.19–537.84) (Table 6B). Two mutations (G → T and A → G) affected enterotoxin-related genes (PJW01_09660 and PJW01_03060), which may collectively influence virulence. Another SNP (G → T) was found in an IS4 family transposase gene (PJW01_01610), suggesting possible genomic rearrangements or ongoing transposition activity.

**Table 6A:** Single-nucleotide polymorphisms identified in *B. thuringiensis* T3 mutant compared to the BLB1 reference genome.

Position	Reference nucleotide	Altered nucleotide	Quality	Affected gene	Product name	Feature type
1843616	T	A	995.65	PJW01_09655	HBL/NHE enterotoxin family protein	CDS
5178095	A	G	362.14	NONE		Intergenic
5282497	A	C	1807.23	PJW01_27745	Hypothetical protein	CDS
5282558	G	A	2052.52	PJW01_27745	Hypothetical protein	CDS
5282578	C	T	1645.56	PJW01_27745	Hypothetical protein	CDS
5667810	T	C	318.85	PJW01_29655	Phage tail protein	Pseudogene
5667871	G	A	255.39	PJW01_29655	Phage tail protein	Pseudogene

**Table 6B:** Single-nucleotide polymorphisms identified in *B. thuringiensis*T8 mutant compared to the BLB1 reference genome.

Position	Reference nucleotide	Altered nucleotide	Quality	Affected gene	Product name	Feature type
285612	G	T	266.19	PJW01_01610	IS4 family transposase	CDS
544327	A	G	290.11	PJW01_03060	Cof-type HAD-IIB family hydrolase	CDS
1844502	G	T	537.84	PJW01_09660	HBL/NHE enterotoxin family protein	CDS
50101882	G	T	272.28	NONE		Intergenic

## 4 Discussion

*B. thuringiensis* is employed as an important microbial bioinsecticide in sustainable agriculture due to its production of insect-specific Cry proteins that are generally safe for non-target organisms. However, the dissemination and environmental persistence of *B. thuringiensis* spores in soil, food, and water raises concerns about their long-term ecological impact (Jouzani et al., 2017; Biggel et al., 2022). Therefore, reducing spore production in *B. thuringiensis* strains is a promising strategy to control the risk of dissemination while maintaining insecticidal activity. Strains with inhibited sporulation allow the reduction of spore persistence in the environment and decrease ecological impact. In this context, classical mutagenesis approaches, such as UV radiation treatment, present an efficient method for generating oligosporogenic mutants (Ghribi et al., 2004). While chemical agents have also been widely used for strain improvement, the present study focused on iterative UV mutagenesis on *B. thuringiensis* BLB1 in order to generate mutants with reduced sporulation capacity. The mutagenesis strategy adopted in this work differs from the multi-agent protocol previously described (Sellami et al., 2026) by focusing exclusively on sequential and iterative rounds of UV irradiation. While our previous study used an optimized Taguchi combination of mutagenic agents to successfully affect the sporulation potency of *B. thuringiensis*, this study employs a refined, single-factor irradiation protocol. This strategy was implemented to facilitate more precise genomic analysis.

In fact, this approach succeeded in producing 12 oligosporogenic mutants complementing and extending previous studies by (Ben Khedher et al. 2011) and (Ghribi et al. 2004) on sporeless strains of *B. thuringiensis* strains. While these studies used nitrous acid, our UV-based mutagenesis strategy offers distinct advantages for strain development. The iterative UV mutagenesis strategy adopted in this study progressively reduced spore production across successive generations of mutants. Additionally, UV treatment can induce deamination of 5-methylcytosine into thymine, a frequent and spontaneous event leading to a GC → AT base pair transition (Coulondre et al., 1978). Unlike single-round mutagenesis, which often results in unstable phenotypes due to DNA repair mechanism, our repeated UV treatments successfully accumulated stable and irreversible defects in sporulation (Hiom, 2003). Furthermore, while all selected oligosporogenic mutants were able to produce delta-endotoxins, their production levels were varied. Mutants T3 and T8 were selected for further investigations, as they demonstrated a significant reduction in spore production associated with a consistent delta-endotoxin production. This combination meets industrial safety requirements and bioinsecticidal efficiency. Following mutants' selection, we examined the effects of these genetic changes on protein production and insecticidal activity. As confirmed by the SDS-PAGE and protein quantification, both T3 and T8 retained measurable levels of delta-endotoxin production compared to the wild-type BLB1 strain, despite their impaired sporulation. To determine whether these differences in protein production resulted in functional changes, insect bioassays were performed.

While BLB1 showed high insecticidal activity against all tested pests, both mutants T3 and T8 exhibited higher LC_50_ values, particularly against *S. exigua* and *S. littoralis*, indicating a modest reduction in insecticidal potency. These observations are consistent with previous reports showing that *Spodoptera* species are generally less susceptible to Cry toxins. (Hernández-Martínez et al. 2008) reported that several Cry proteins induce limited mortality but still affect larval performance in *S. exigua*, highlighting the complexity of toxin host interactions. Moreover, both mutants exhibited LC_50_ values against *O. nubilalis, G. molesta*, and *E. kuehniella* that were comparable to those of the wild-type strain. These results indicate that, despite their reduced sporulation and reduced Cry protein levels, these mutants retain their toxicity and efficiency against these pests. Further studies will be conducted to investigate the underlying mechanisms of the differential toxicity observed between mutants T3 and T8 and the wild-type BLB1 by comparing their specific mode of action, including protoxin activation by midgut proteases and the binding affinity of toxins to Brush Border Membrane Vesicles (BBMV).

Interestingly, these findings confirm that both mutants maintained functional *cry* genes, suggesting that toxigenic capacity was not completely compromised. This differential effect of the strong reduction in sporulation and the maintained toxin production levels highlights the industrial relevance of random mutagenesis for generating oligosporogenic strains (Ben Khedher et al., 2011).

To determine whether the observed phenotypic changes were associated with growth dynamics and nutrient consumption, we analyzed the growth kinetics and glucose consumption of *B. thuringiensis* BLB1 and its mutants T3 and T8. This analysis revealed distinct differences in sporulation timing that were correlated with glucose depletion patterns. In BLB1, vegetative growth proceeded rapidly during the first 12–16 h, concurrent with a sharp decline in glucose concentration. Sporulation initiated early at 18 h and reached a maximum by 30–36 h which was consistent with the canonical four-phase developmental cycle described for *B. thuringiensis* var. *israelensis*, where nutrient depletion, particularly glucose, serves as the primary trigger for sporulation (Berbert-Molina et al., 2008). In contrast, spore production in T3 and T8 showed a significant delay, with a progressive increase extending to 48 h and reaching stabilization after 72 h, suggesting impaired sporulation. These findings highlighted that although mutants maintained vegetative growth, their metabolic activity, reflected by delayed sporulation and glucose consumption, was reduced compared to the wild-type strain. This behavior could be associated with mutations affecting sporulation signaling or carbon flux regulation. Similar delays in sporulation under high glucose concentrations have been reported in other *B. thuringiensis* strains, where excess or imbalanced nutrient conditions disrupted the normal progression of developmental phases (Berbert-Molina et al., 2008). The observed delay in T3 therefore points to a decoupling between glucose depletion and sporulation onset, highlighting that sporulation in this mutant is not synchronized with substrate exhaustion but possibly influenced by altered intracellular regulatory networks. To identify the genetic basis of these phenotypic changes, WGS was performed. Annotation of the selected mutants (T3 and T8) confirmed that the mutagenesis was primarily induced through point mutations and small insertions or deletions. Comparative genomic analysis against the wild-type BLB1 reference revealed that both mutants preserved the overall chromosomal backbone and the plasmid encoding *cry* genes. Notably, although the T8 mutant displayed the lowest level of cry gene expression, it retained toxicity against tested larvae. This suggests that the maintained toxicity of T8 may instead be attributed to the combined action of the remaining *cry* genes together with other virulence factors identified in its genome, such as chitinase and metalloprotease genes. These proteins facilitate midgut barrier disruption by enhancing toxin penetration, which may compensate the reduced Cry protein levels and contribute to the overall insecticidal efficacy of the mutant (Rabha et al., 2023).

Additionally, none of the detected mutations was found in *cry* genes, which may account for the preserved Cry protein expression and insecticidal activity. Moreover, mutations other cellular pathways may be affected, which may be explained by the altered sporulation kinetics and glucose consumption patterns observed in the mutants. In particular, the SNP identified in the T8 strain affected a transposase-encoding gene, suggesting the potential genomic instability and genome disruption rather than targeted effects on toxin genes (Siguier et al., 2014). This may support the atypical behavior of T8, which retained normal growth, Cry protein production, and toxicity against all tested pests, while exhibited distinct metabolic and kinetic profiles. Together, these results indicate that the introduced mutations could influence regulatory and metabolic processes without affecting the insecticidal capacity of the strain. Moreover, despite the low overall genome coverage of the T3 mutant, all target genes relevant to this study, such as key virulence-associated genes and also the genes affected by mutagenesis, were successfully identified and characterized, which were sufficient to address the objectives of this work. Finally, although the complete reconstruction of these genomes was not achieved, the sequencing data generated were sufficient for the required genetic characterization and fulfilled the analytical requirements of the present study.

Overall, the selected mutants demonstrated strong potential as oligosporogenic and effective insecticidal candidates, combining reduced sporulation with maintained toxicity against agricultural pests, a feature that is particularly advantageous for limiting spore dissemination in commercial biopesticide applications.

## 5 Conclusion

This study demonstrates that iterative UV mutagenesis is an effective strategy for generating *B. thuringiensis* oligosporogenic mutants while retaining strong biocontrol activity. Specifically, T3 and T8 mutants exhibited a clear decrease in spore production, retained protein expression and toxicity against target insect pests including *G. molesta* and *O. nubilalis*. Despite the oligosporogenic phenotype of these mutants, genomic analysis confirmed the absence of genetic variations in genes encoding Cry proteins as well as in key genes involved in the sporulation process. These findings make these strains promising candidates for environmentally safer biological control applications.

## Data Availability

The genome of BLB1 has been previously sequenced, and its data is publicly available under the NCBI BioProject accession number PRJNA924104 with raw sequences reads available under the accession number SRR24475419. Moreover, genome sequencing data of the mutants generated in this study have been deposited in the National Center for Biotechnology Information database under BioProject accession number PRJNA1417242. Raw sequencing reads are available in the Sequence Read Archive under accession number SRR37072234 and SRR37541361.
